# Armored eyes of the whale shark

**DOI:** 10.1371/journal.pone.0235342

**Published:** 2020-06-29

**Authors:** Taketeru Tomita, Kiyomi Murakumo, Shinya Komoto, Alistair Dove, Masakatsu Kino, Kei Miyamoto, Minoru Toda

**Affiliations:** 1 Okinawa Churashima Research Center, Okinawa Churashima Foundation, Motobu-cho, Okinawa, Japan; 2 Okinawa Churaumi Aquarium, Okinawa Churashima Foundation, Motobu-cho, Okinawa, Japan; 3 Research Support Division, Imaging Section, Okinawa Institute of Science and Technology Graduate University, Tancha, Onna-son, Okinawa, Japan; 4 Georgia Aquarium, Atlanta, Georgia, United States of America; Consejo Nacional de Investigaciones Cientificas y Tecnicas (CONICET), ARGENTINA

## Abstract

This report elaborates on adaptations of the eyes of the whale shark *Rhincodon typus* (Elasmobranchii, Rhincodontidae), including the discovery that they are covered with dermal denticles, which is a novel mechanism of eye protection in vertebrates. The eye denticle differs in morphology from that of the dermal denticles distributed over the rest of the body, consistent with a different function (abrasion resistance). We also demonstrate that the whale shark has a strong ability to retract the eyeball into the eye socket. The retraction distance was calculated to be approximately half the diameter of the eye, which is comparable to those of other vertebrates that are known to have highly retractable eyes. These highly protective features of the whale shark eye seem to emphasize the importance of vision for environmental perception, which contradicts the general, though poorly established, notion of low reliance on vision in this species.

## Introduction

The eye, which is the organ that obtains optical information from the external environment, must be located on or near the surface of the body. Thus, eyeballs face a potential risk of damage from mechanical, chemical and biological hazards. Many terrestrial and aquatic vertebrates prevent this risk by the coverage of eye surfaces with eyelids [1]. For example, carcharhinid and sphyrnid sharks have nictitating membranes or “third eyelids”, which cover their eyes completely during their feeding activities [2]. The outer surface of this membrane is covered with dermal denticles, which likely increases its protective ability [3]. In contrast, many other elasmobranchs that are not equipped with nictitating membranes have to protect their eyes in different ways, such as retracting the eyeballs into the head (e.g., electric ray [4]; guitarfish [5]), or rotating the eyeballs back into the orbit (e.g., white shark [6]). However, the eye-protection mechanism in many elasmobranch species remains largely uninvestigated.

**Fig 1:**
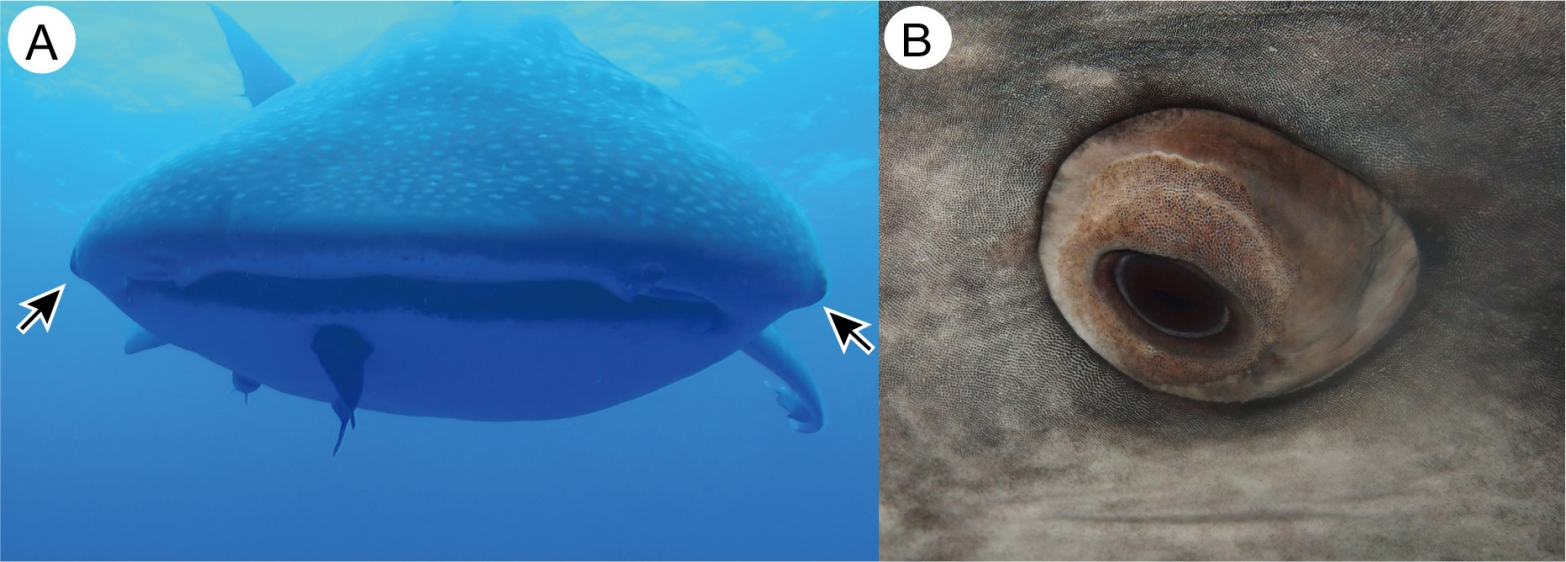
Eyes of the whale shark. A. Anterior view of the whale shark, showing the locations of the eye (arrows). Note that whale shark eye is well projected from the orbit. Photo was taken in the sea near Saint Helena Island. B. Close-up view of the left eye of a captive whale shark (Specimen A).

We examined the eye protection mechanism of the whale shark *Rhincodon typus* (Elasmobranchii, Orectolobiformes, Rhincodontidae). The whale shark is the largest fish, reaching over 18 m in total length [7]. Its eyes are located at the antero-lateral corner of the roughly square-shaped head, and are considerably projected from the orbit (Fig 1). These features are expected to increase the risk of injury to the eyes, during swimming through drifting/floating objects in water, for example. As far as we know, the only description about the eye protection mechanism of the whale shark is found in Martin [8]. He reported, as a personal observation, that the whale shark protects its eyes by rotating the entire eyeball back into the eye socket. However, no objective data were provided in the paper.

Recent success in the care and maintenance of whale sharks in aquariums made it possible to study the eye protection mechanism in this species (e.g., Matsumoto et al. [9]). The paucity of information on the eye protection mechanism of the whale shark may be attributed to the extremely low access to live specimens. In general, studies on large aquatic vertebrates are difficult because of their small population size, strong swimming ability, and pelagic habitat. Thus, most studies on these animals have been based on a small number of dead specimens [10]. We aimed to describe, for the first time, the detailed kinematic and morphological features of whale shark eyes that are associated with eye protection. We did this by applying some recent techniques, such as underwater sonography and micro-computed tomography, to analyze both live and dead specimens, and to compare them with those of other elasmobranchs.

## Materials and methods

Animal handing during ultrasound was done in strict accordance with the guidelines for animal experiments of the Okinawa Churashima Foundation, with the same consideration for animal care and welfare as that for “higher” vertebrates (reptiles, birds, and mammals). However, as the guidelines stipulated, the approval from the Institutional Animal Care and Use Committee of Okinawa Churashima Foundation, required for higher vertebrates, is waived for “lower” vertebrates including fishes.

### Morphology

Morphological observation was conducted on the formalin-preserved eyeball of a whale shark (OCF-F04248) in the collection at the Okinawa Churashima Research Center (Okinawa, Japan). This eyeball was extracted from a dead whale shark specimen during a dissection conducted by the aquarium staff at the Okinawa Churaumi Aquarium in 2017. This specimen was originally caught as a bycatch in a net set by local fishermen in Okinawa, Japan, and was donated to the Okinawa Churaumi Aquarium for scientific purposes. The anteroposterior diameter of the eyeball was 65.0 mm.

Computed tomography (CT) data were acquired from this specimen using a micro CT scanner (Zeiss Xradia 510 Versa; Zeiss, Oberkochen, Germany) at an X-ray setting of 50 kV, equipped at the Okinawa Institute of Science and Technology (Okinawa, Japan). Three-dimensional reconstructions were prepared using IMARIS software (Bitplane, Zürich, Switzerland). The voxel size of the three-dimensional data was 18.2 μm for the observation of the entire eyeball morphology and 5.2 μm for the observation of the more detailed structure (e.g., morphology of each denticle). Terminology of denticle morphology follows Thies and Leidner [11]. The functional categories of shark denticles referred to in this study were based on Reif [12], in which shark denticles were divided into four groups: defense, protection from abrasion, bioluminescence, and the reduction of hydrodynamic drag.

### Kinematics

Two captive whale sharks (specimens A and B) were used for the external kinematical observations. Specimen A (male, 8.7 m in total length) and specimen B (female, 8.1 m in total length) have been maintained in an exhibition tank at the Okinawa Churaumi Aquarium (Okinawa, Japan) since 1996 and 2012, respectively. One of the authors (KiM) swam with these specimens, and eye movement was recorded from approximately 30 cm away from the eye surface using a GoPro HERO5 video camera (GoPro, Inc., San Mateo, CA).

Ultrasound experiments were also conducted on two captive whale sharks (specimens A–C). Specimens A and B were the same individuals that were used in the external kinematical observation described above, and specimen C (male, 5.54 m in total length) was maintained in an open water fish pen near the Okinawa Churaumi Aquarium. A portable ultrasound device with pressure- and water-proof housing [13] was used to document eye retraction (ARIETTA Prologue; Hitachi-Aloka Medical Ltd., Tokyo, Japan). The transducer of the ultrasound was held 0.5 cm away from the surface of the eyeball, and acquired the ultrasound footage during eye retraction. This is possible because water conducts the ultrasound signal well, and direct contact with the skin is not required as it is in terrestrial animals.

## Results

### Morphology

**Fig 2:**
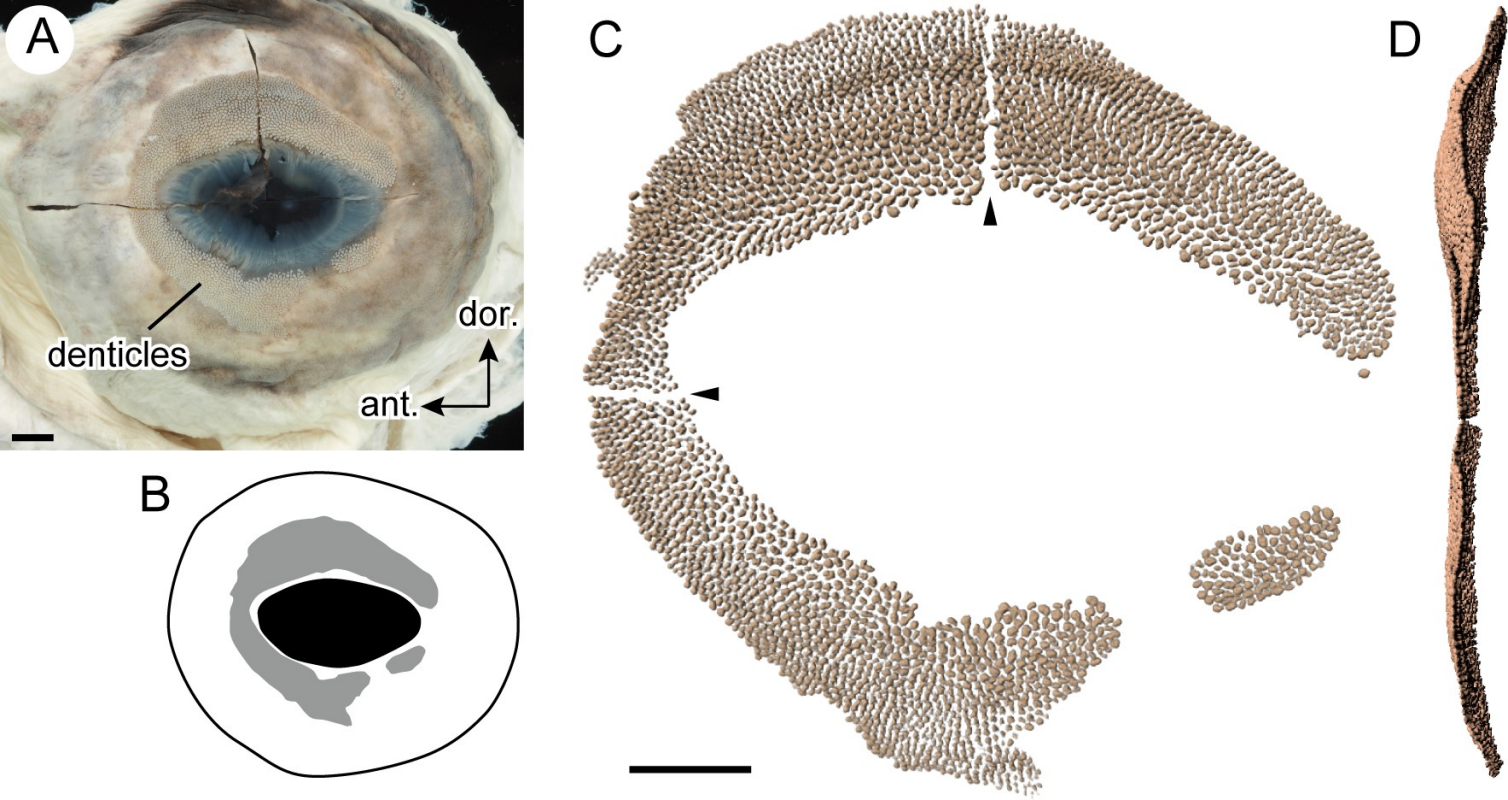
Eye denticles of the whale shark. A. Distal view of left eyeball (OCF-P04248). B. Line drawing of OCF-P04248, showing the distribution of the eye denticles (gray area). C. Three-dimensional image of eye denticle aggregation obtained from computed tomography data. Horizontal and vertical lines of no denticle area (arrowheads) are artifacts. D. Posterior view of panel C. Scale bars = 0.5 cm.

Numerous dermal denticles are distributed on the eye surface around the iris (referred to as eye denticles, Fig 2). According to the object-counting option in the IMARIS software, the total number of eye denticles was ca. 2900 in OCF-P04248.

**Fig 3:**
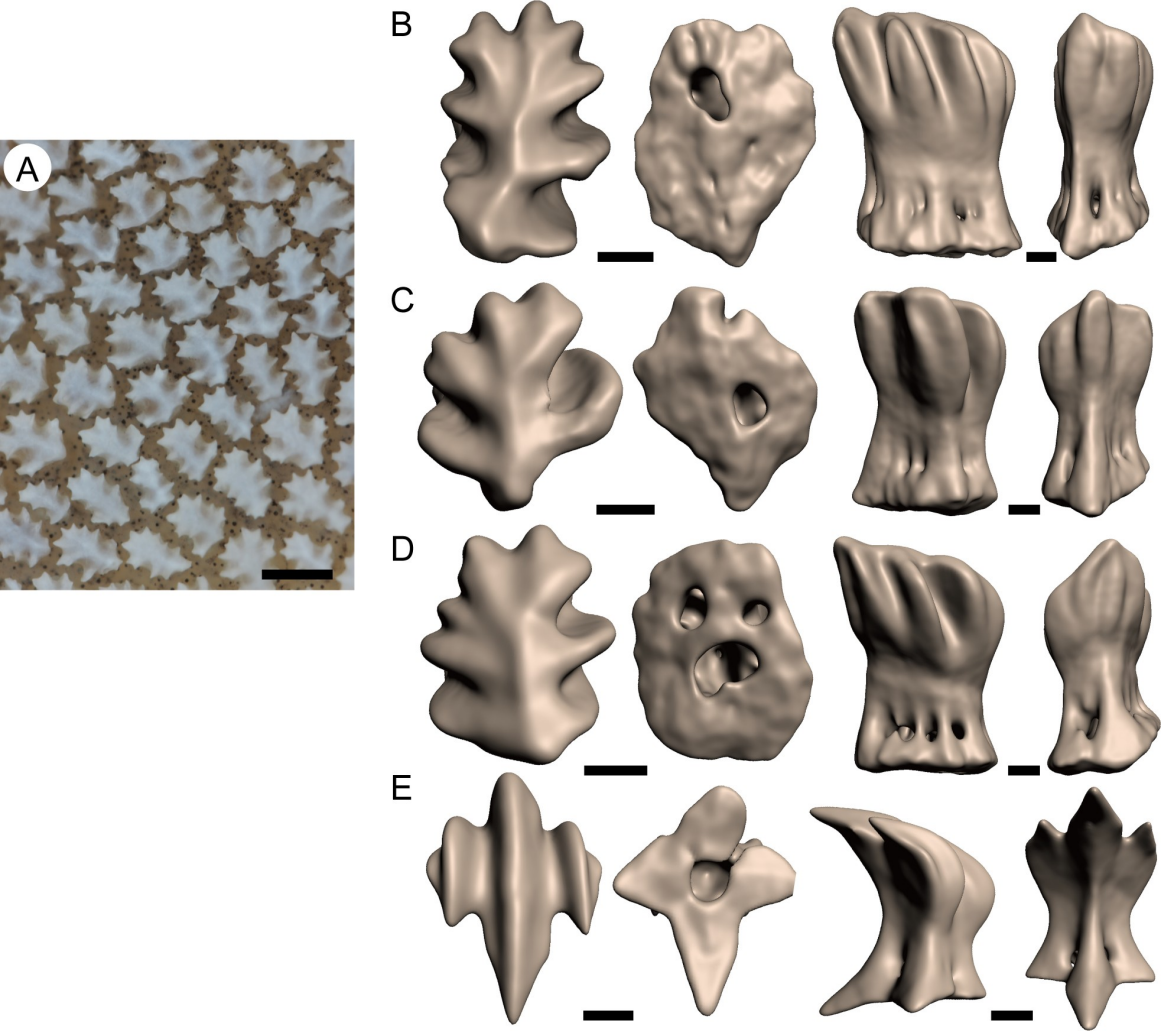
Morphology of each eye denticle of the whale shark. A. Close-up of aggregated eye denticles. B–D. 3D reconstruction of eye denticles obtained from computed tomography data, showing the morphological variations. E. Dermal denticle from the skin just above of the eyeball. In B–E, apical, basal, lateral, and posterior views from left to right. Scale bars = 500 μm in A and 100 μm in B–E. See supporting information S1 Video.

The general morphology of each eye denticle is characterized by a central ridge running through the longitudinal axis of the denticle and several (usually 6–8) sub-ridges branching laterally from the central ridge (Fig 3A–3D and S1 Video). Due to these ornamentations, the eye denticle presents an oakleaf-like appearance from the apical view. The base of the eye denticle is oval-shaped in basal view and comprises multiple (generally more than 8) foramina of the neck canal in the lateral view. These features are in contrast to the denticles distributed over the rest of the body that have triple ridges arranged in parallel on the apical surface of the crown, rectangle-shaped base in basal view, and 4 foramina of neck canal in the lateral view (Fig 3E).

### Kinematics

**Fig 4:**
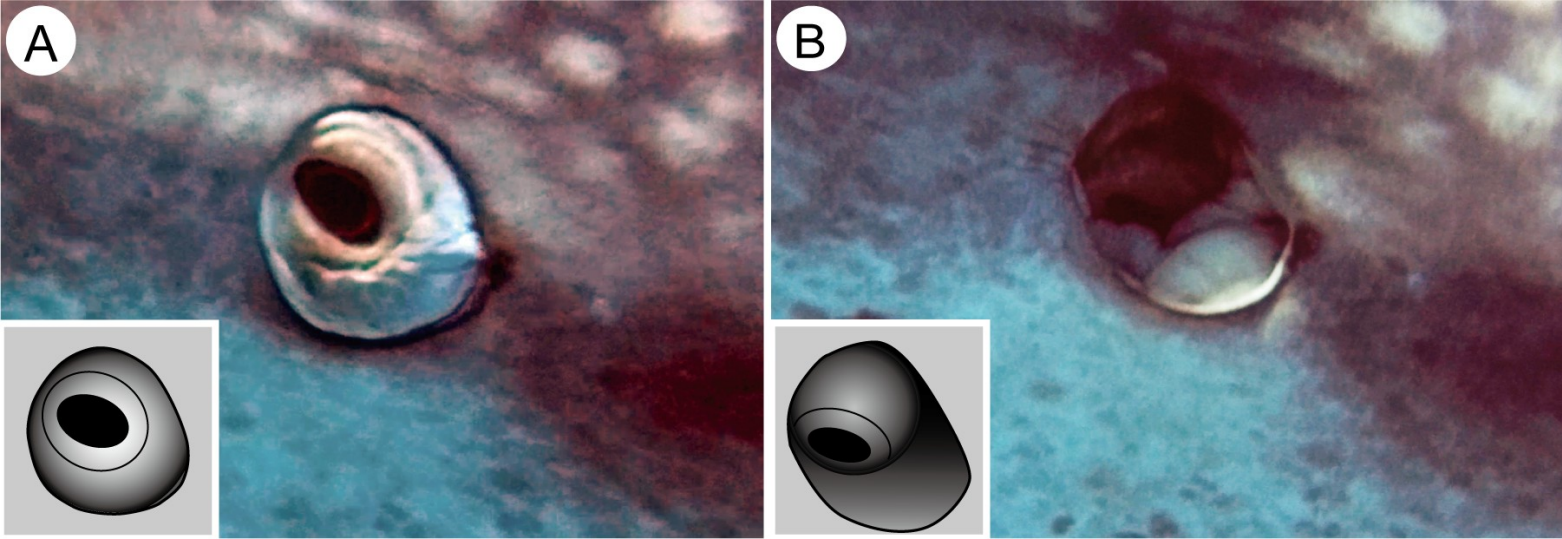
Eye retraction of the whale shark (Specimen A). Relaxed (A) and retracted (B) phases of the whale shark eye. The lower left of each panel is a schematic diagram showing the location and rotation of the eyeball. See supporting information S2 Video for original video clip.

**Fig 5:**
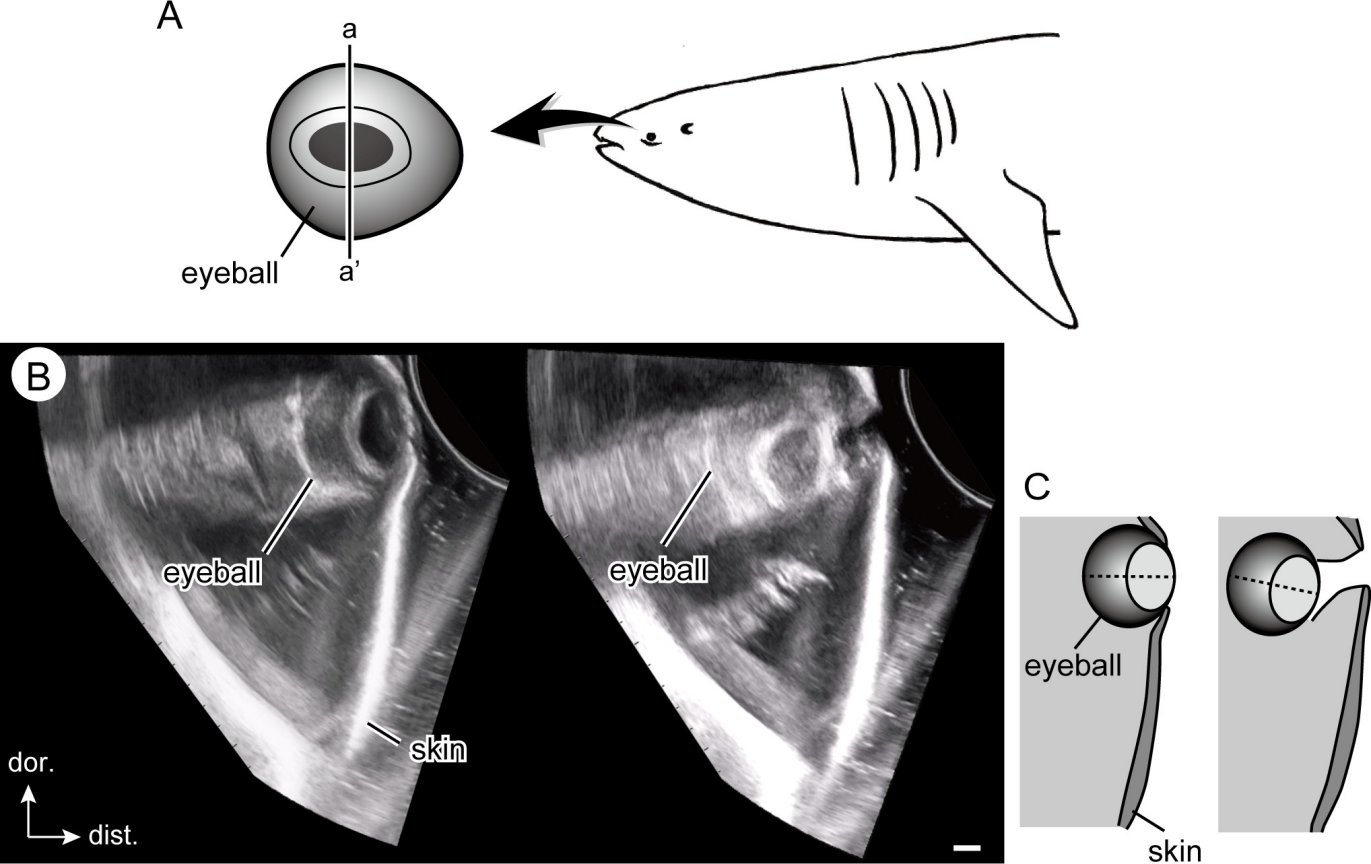
Eye retraction of the whale shark in ultrasound data. A. Left lateral view of the whale shark head, showing the location where the ultrasound data was obtained in this study. Ultrasound data are shown in the cross section passing through the maximum height of the eyeball (a–a’). B. Relaxed (left) and retracted (right) phase of the whale shark eye (specimen C). Scale bar = 1 cm. C. Schematic illustration of panel B. Dotted line represent the distal-proximal axis of the eyeball. See supporting information S3 and S4 Videos for original video clips.

Eye retraction behavior was observed in all whale sharks (specimens A–C) examined in this study (Fig 4 and S2 Video). When an object approached (in this case the diver), the eyes were retracted into the orbit with a duration (from the onset of eye retraction to complete retraction) of less than 1 s. Ultrasound data showed that the maximum retraction distance was 3.3 and 2.8 cm (50.4 and 49.8% of the eyeball diameter) in specimens A and C, respectively (S3 and S4 Videos). During retraction, the eyeball rotated ventrally (Figs 4 and 5), and white connective tissue from the retrobulbar space was displaced around the eyeball and partially filled the space where the eye used to be (Fig 4B and S2 Video). In general, eye retraction occurs for a short duration only when an object approaches the eyes of a specimen. However, longer-term eye retraction behavior was observed once in a captive specimen (female, 5 m in total length at the time) at the Georgia Aquarium, which kept its eyes retracted for approximately 10 days in June, 2006, immediately following its transport to Atlanta from Taiwan.

## Discussion

Our study confirmed that the eyes of the whale shark are covered with eye denticles. In general, the superficial layer of the elasmobranch eye, like in other vertebrates, consists of two specialized epithelial tissues, the cornea on the iris region, and the conjunctiva on the outer iris region [1]. Considering that these tissues are exposed and that whale sharks lack eyelids; the eye surface is less protected from mechanical damage than other regions of the body that are covered with mineralized dermal denticles. Thus, the covering of the eye surface with denticles in the whale shark is probably useful in reducing the risk of mechanical damage to the eye surface. As far as we know, eye denticles have not been found in other elasmobranchs, including species closely related to the whale shark, such as the tawny nurse shark (*Nebrius ferrugineus*) and the zebra shark (*Stegostoma fasciatum*). It seems likely, therefore, that eye denticles are a characteristic unique to the whale shark. It should be noted that eye armor was previously described in some fossil chondrichthyans. Dean [14] reported that a Paleozoic *Cladoselache* has ring-like plates around the iris (“circum-orbital plates”), and assumed that this structure was derived from dermal denticles. However, this structure is now reinterpreted as a sclerotic ring, not a denticle-derived structure [15].

Denticles on the eye are morphologically distinct from denticles distributed over the rest of the body. The apical surface of the eye denticle is ornamented with laterally branched ridges, resulting in an oakleaf-like appearance. Interestingly, a similar morphology is found in the body denticles of the horn sharks, *Heterodontus* spp. [16]. A previous study classified the morphology of shark denticles into four groups based on presumed function: defense, protection from abrasion, bioluminescence, and hydrodynamic drag reduction [12]. Following this classification system, the denticle of the horn sharks is a typical example of a protection-from-abrasion-type denticle [12]. Morphological similarity between eye denticles of the whale shark and body denticles of the horn shark supports our hypothesis that the main function of the eye denticle is mechanical protection. The denticles on the rest of the whale shark’s body, such as the head, trunk and fins, are characterized by parallel, triple ridges on the upper surface, presenting a drag-reduction type morphology (Fig 3E). Interestingly, the denticles that cover the nictitating membranes of carcharhinid and sphyrnid sharks have also been thought to play a role in eye protection [3]. Though the overall morphologies of the denticles in the nictitating membranes of carcharhinid and sphyrnid sharks are different from those of whale sharks, they both have especially thick denticle crowns, which suggests that they have a similar function of mechanical protection.

The present study also revealed that the eye protection mechanism of the whale shark involves active eye retraction with partial ventral rotation. Maximum retraction distances were calculated to be approximately half (49.8% and 50.4%) of the eyeball diameter. These values are comparable to vertebrates with highly retractable eyes, such as the northern leopard frog *Rana pipiens* (50%, calculated by X-ray [17]) and bottlenose dolphin *Tursiops* sp. (40%–60% [18]). The batoid giant guitarfish (*Rhynchobatus djiddensis*) demonstrates the highest eye retraction of any vertebrate, at 101% [5]. Among elasmobranchs, eye retraction ability has been well described in batoids, but not in sharks. As far as we know, the only published record of eye retraction behavior in sharks is seen in the supplementary video footage (S4 Video) in McNeil et al. [19]. This shows that the sixgill shark (*Hexanchus griseus*) retracts the eyeball during biting behavior. However, eye retraction distance could not be calculated from this footage. It is likely that whale sharks maintain their vision during eye retraction because the pupils of the whale sharks in this study were not completely covered with surrounding white tissues when their eyes had retracted, though their visual field would be much more restricted than when their eyes are positioned normally. In fact, the animal that kept its eyes retracted for approximately 10 days at the Georgia Aquarium appeared to have no problem navigating the exhibit space, until its eyes returned to their normal positions suddenly and, apparently, spontaneously.

Our data also revealed that eye retraction in the whale shark is accompanied by eye rotation. Eye rotation ability of the whale shark was previously documented in a study by Martin [8], although the rotating direction is different between his and our observations (rotating posteriorly in a study by Martin [8]; ventrally, in the present study). Eye retraction and rotation together was also observed in the sixgill shark [19].

The present study provides novel information on the visual capacity of the whale shark. Because of the relatively small eyes (eye diameter less than 1% of total length [2]), it has been assumed that the whale shark depends little on vision compared with other senses such as olfaction [20]. This notion was supported by the small proportional size of the midbrain (mesencephalon), which is a division of the brain that is responsible for processing the visual information [21]. However, the highly protected features of the whale shark eye, in contrast to the traditional view, seems to suggest the importance of vision in this species. Interestingly, Martin [8] showed that whale shark eyes actively track divers swimming 3–5 m away from the animal, suggesting that vision of the whale shark plays an important role in short-range perception. Such active visual tracking is also seen in the captive whale sharks at Okinawa Churaumi Aquarium (KiM, pers. obs.) and at the Georgia Aquarium (AD, pers. obs.). Future research should focus on the optical sensory capacity (e.g., visual field, acuity, color range, and sensitivity) of the whale shark vision system.

## Supporting information

S1 Video 3D reconstruction of eye denticle obtained from computed tomography data. (MP4)

S2 Video Eye retraction of Specimen A. (MP4)

S3 Video Ultrasound footage of eye retraction in Specimen A. (MP4)

S4 Video Ultrasound footage of eye retraction in Specimen C. (MP4)
